# Unveiling the neutrophil-Notch2-ISC axis: asiatic acid’s therapeutic strategy in infectious colitis

**DOI:** 10.3389/fimmu.2025.1634063

**Published:** 2025-09-22

**Authors:** Wenshu Zou, Minyong Zhong, Zerong Pei, Yuxin Chen, Wenwen Deng, Hui Li

**Affiliations:** ^1^ Jiangxi Province Key Laboratory of Traditional Chinese Medicine Pharmacology, Institute of Traditional Chinese Medicine Health Industry, China Academy of Chinese Medical Sciences, Nanchang, China; ^2^ Postdoctoral Fluxion Station, China Academy of Chinese Medical Sciences, Beijing, China; ^3^ Jiangxi Province Key Laboratory of Traditional Chinese Medicine Pharmacology, Jiangxi Health Industry Institute of Traditional Chinese Medicine, Nanchang, China; ^4^ Institute of Chinese Materia Medica, China Academy of Chinese Medical Sciences, Beijing, China

**Keywords:** salmonella, asiatic acid, neutrophil, intestinal stem cell, notch pathway

## Abstract

**Introduction:**

*Salmonella*-induced colitis is a global health burden characterized by intestinal barrier disruption and deficient epithelial repair that involves a critical interplay between neutrophil dynamics and intestinal stem cell (ISC) regeneration. Current therapies do not target this interplay and do not adequately address therapeutic need. This study investigated the therapeutic mechanism of asiatic acid (AA) in a murine *Salmonella typhimurium* (*S.T*) infection model, focusing on its effects on the neutrophil-Notch-ISC axis.

**Methods:**

Balb/c mice were administered *S.T* for 3 days to model *S.T* infection AA (10 mg/kg) was gavage administered to mice 6 h after the *S.T* infection. Neutrophil-deficient mice were generated by daily intraperitoneal injection of Ly6G for 3 days.

**Results:**

Mouse colons were analyzed histologically, and transcriptomic, network pharmacology, western blotting, and immunohistochemistry investigations were performed. AA restored mucosal integrity by upregulating tight junction proteins (occludin and claudin 1) and acidic mucin granules levels, and by rescuing ISC proliferation through suppression of Notch2/Hes5/Hey1 signaling. Multiomics analyses further revealed the modulation of neutrophil chemotaxis and inflammatory pathways by AA. Strikingly, neutrophil ablation reduced the efficacy of AA, confirming that AA acts via neutrophil-mediated containment of bacterial invasion and epithelial shedding.

**Discussion:**

By revealing the neutrophil-Notch2-ISC axis as a pivotal regulator of mucosal repair, our findings show AA to be a dual-action therapeutic agent that synergizes immune containment and regenerative pathways. These findings highlight an aspect of *S.T* pathogenesis and underscore the potential of natural compounds to harmonize host defense and tissue regeneration, offering a transformative strategy for infectious colitis beyond conventional anti-inflammatory approaches

## Introduction

*Salmonella*-induced colitis poses a grave threat to global public health, with the World Health Organization estimating 200 million cases and over 200,000 fatalities annually (1, 2). Gastroenteritis is the predominant clinical manifestation of *Salmonella* infection and is often accompanied by disruption of the intestinal mucosal barrier (3–5). Intestinal epithelial cells (IECs), the frontline defenders of mucosal integrity, prevent pathogen invasion into submucosal tissues (6, 7). EPCAM, a key molecule of IEC intercellular adhesion, is downregulated during the *Salmonella* infection, resulting in weakened adhesion (8). IECs that have lost their adhesive capacity are expelled into the intestinal lumen and degraded (9). However, *Salmonella* can form *Salmonella* cysts within epithelial cells, and the levels of EPCAM in these epithelial cells are not affected, which is conducive to bacterial replication and invasion (10, 11). Concurrently, infection reduces the levels of tight junction proteins (e.g., occludin, claudin 1) and activates pro-inflammatory pathways, compounding tissue damage and impairing epithelial repair (11, 12). Intestinal stem cells (ISCs), located in crypt bases, regenerate IECs to maintain barrier homeostasis (13). Notably, *Salmonella* infection suppresses ISC markers (e.g., LGR5), impairs IEC differentiation, and exacerbates mucosal injury (14–16). The Notch signaling pathway, a key regulator of ISC fate, drives ISC proliferation and lineage specification (17, 18). Mice deficient in Notch1/Notch2 exhibit defective ISC regeneration, underscoring its centrality in epithelial repair (19). Emerging evidence further implicates immune cells and their cytokines in modulating ISC activity during inflammation (20, 21). Neutrophils, as important responders, infiltrate the intestinal lumen post-infection and regulate IEC shedding (22, 23). Paradoxically, their recruitment of secondary immune cells may suppress ISC differentiation through inflammatory mediators (24, 25). Despite these observations, direct evidence linking neutrophil dynamics to mucosal barrier restoration in *Salmonella* colitis remains elusive, hindering the development of therapies targeting immune-stem cell crosstalk.

The pathogenesis of *Salmonella* infection and its interplay with intestinal homeostasis have been extensively studied, with mechanisms of IEC invasion, intracellular survival, and microbiota-immune crosstalk during infection being proposed (9, 10). Some studies have explored how *Salmonella* invades IECs and survives and replicates in cells (26, 27). Other studies have focused on the interaction between intestinal microbiota and *Salmonella* infection, and the immune response of immune cells during infection (28, 29). Recently, natural products for the treatment of colitis have been explored; for example, the potential of extracts from plants, such as *Centella asiatica*, for the treatment of inflammatory bowel disease has attracted much attention (30, 31). The active ingredient in *C. asiatica* extract, asiatic acid (AA), has a therapeutic effect against dextran sulfate sodium-induced colitis, reducing weight loss and colon shortening, and lowering the activation of nuclear factor kappa B (NF-κB) and the levels of downstream pro-inflammatory factors, tumor necrosis factor (TNF)-α and interleukin (IL)-6 (32, 33). In addition, AA exerts its antibacterial effect by destroying bacterial biofilms and inhibiting bacterial adhesion and invasion, including for *Salmonella typhimurium (S.T)*, *Staphylococcus aureus*, and *Pseudomonas aeruginosa* (34–36). However, the specific effect and mechanism by which AA alleviates *Salmonella*-related intestinal damage is still unclear. These gaps in understanding hinder the development of targeted therapies for *Salmonella* colitis, underscoring the need to fully elucidate AA’s mechanism of action within the context of neutrophil-ISC crosstalk.

We have examined the multi-layered mechanism by which AA alleviates *S.T*-induced colitis. Using a murine *Salmonella* infection model coupled with neutrophil depletion (using an anti-Ly6G antibody), we systematically assessed the impact of AA on colon morphology, barrier integrity (via the levels of tight junction proteins and acidic mucins granules), and ISC activity. Integrated network pharmacology, western blotting, immunohistochemical analyses, and transcriptome sequencing revealed that AA restores ISC proliferation by suppressing Notch2 pathway activation (Notch2/Hse5/Hey1) and modulating neutrophil recruitment. Critically, the protective effects of AA, including barrier restoration and attenuation of injury, were reduced in neutrophil-depleted mice, demonstrating its dependence on neutrophil-mediated immune regulation. These findings reveal a novel “neutrophil-Notch2-ISC” axis through which AA repairs intestinal barrier dysfunction and suggest a targeted therapeutic strategy for infectious colitis. Furthermore, our study highlights the potential of natural compounds to modulate immune-stem cell interactions.

## Methods

### Mice infections

Male and female specific pathogen-free (SFP) BALB/c mice aged 6–8 weeks with different microbiota complexity were purchased from Beijing SiPeiFu Biotechnology Co. and kept at 23 ± 2°C under a 12 h light/dark cycle with *ad libitum* access to food and water. Mice were pretreated with 25 mg streptomycin by oral gavage 24 h prior to infection. *S.T* ATCC 14028 were grown overnight in lysogeny broth medium at 37°C with shaking at 220 rpm and then cultured (1:33) in fresh lysogeny broth medium for 3–3.5 h. To model *S.T* infection, mice were administered *S.T* by oral gavage (1 × 10^9^ CFU/mouse). Six hours after *S.T* gavage, mice were administered AA (Aladdin, Shanghai, China, CAS: 464-92-6, C_30_H_48_O_5_, HPLC ≥ 98%) by oral gavage (10 mg/kg) on 3 consecutive days. The infected mice were euthanized 72 h after infection. The sample-size was not predetermined, and mice were randomly assigned to groups. Neutrophils were depleted in mice by daily intraperitoneal injection of anti-mouse Ly6G-Purified (Cat No: L280, Leinco Technologies, USA) (250 μg/mouse) for 3 days.

All animal experiments were approved by the Committee on Animal Care and Use of the Institute of Traditional Chinese Medicine Health Industry (licence numbers: SYXK(Gan)2023-0008).

### Histopathological examination

Intestinal tissue from the anus to the cecum was isolated from mice. Colon tissues were fixed in 4% formaldehyde, embedded in paraffin, and sectioned for staining with hematoxylin-eosin (H&E) (Servicebio, Beijing, China) or Alcian blue/periodic acid-Shiff (AB/PAS) solutions. Sections were observed with an SV200 microscope (Olympus, Japan).

Acidic mucin granules (blue) density was calculated using Jmage J software as follows: Open image → Image → Color → Segmentation Channel → Select Blue → Adjustment → Threshold → Apply → Analyze → Measurement → Acid Mucin Density (%Area).

### Western blotting

Based on a previously described method (37), samples were prepared from colon tissues using RIPA buffer (KeyGEN BioTCHE, Nanjing, China). Total proteins were extracted, and protein concentration was measured using a BCA kit. Equal amounts of proteins were separated by 10%–12% sodium dodecyl sulfate polyacrylamide gel electrophoresis and were then transferred onto nitrocellulose membranes (Cytiva, USA). After blocking with 5% skim milk for 1 h, the membranes were incubated with the indicated primary antibodies at 4°C overnight. The main primary antibodies used are as follows: Occludin (Cat No. 27260, 1:1000, Proteintech, Wuhan, China), Claudin1 (Cat No. 28674, 1:500, Proteintech), β-actin (Cat No. 81115, 1:1000, Proteintech), Hes5 (Cat No. 22666, 1:200,:1000, Proteintech), Hey1 (Cat No. 19929,:1000, Proteintech), Notch2 (Cat No. 5723, 1:1000, CST), Ly6G (Cat No. 14-5931-82, 1:1000, Thermo Fisher Scientific, USA), LGR5 (Cat No. PA5-87974, 1:1000, Thermo Fisher Scientific). The visualization of the protein bands was completed with ECL kit (Advansta, USA).

### Immunohistochemistry

Three-micron-thick sections were prepared from wax-embedded tissues, deparaffinized in xylene and alcohol, incubated with EDTA at 100°C for 20 min, 3% hydrogen peroxide at room temperature for 25 min, and finally with 5% bovine serum albumin at room temperature for 1 h. Sections were then incubated sequentially with primary and secondary antibodies. The following antibodies and dilutions were used for the staining of different samples: EPCAM/CD326 (Cat No. 21050, 1:200, Proteintech), 1:200 *S.T*-LPS (Cat No. MA1-83451, 1:200, Thermo Fisher Scientific, USA), Ly6G (Cat No. 14-5931-82, 1:100, Thermo Fisher Scientific, USA), LGR5 (Cat No. PA5-87974, 1:100, Thermo Fisher Scientific) combination with the respective secondary antibodies, anti-Rabblt AlexaFluor™ 488 (Cat No. A11008, 1:500, Thermo Fisher Scientific), anti-Mouse AlexaFluor™ 488 (Cat No. A11011, 1:500, Thermo Fisher Scientific), Cyanine3 (Cat No. A10521, 1:400, Thermo Fisher Scientific), and DAPI (Beyotime Biotechnology, Shanghai, China). Images were observed with an SV200 microscope (Olympus, Japan).

### Phagocytosis by neutrophils

HL-60 cells were stimulated into neutrophils with 1 μM all-trans-retinoic acid, then cultured in 96-well plates. Each well was infected with *S.T* at a multiplicity of infection of 10. AA (50 and 100 μM) was added to the culture immediately after *S.T* infection. Following a 3 h infection with *S.T*, HL-60 cells were lysed with sterile distilled water for 5 min. The cell lysates were then diluted and plated on LB agar and incubated at 37°C for 24 h, permitting an estimation of the CFUs for *S.T.*

Phagocytosis (%) = (Counts of released bacteria after Triton treatment/Counts of added bacteria) * 100.

### Bacterial growth measurements

Bacteria were grown to early exponential phase in Luria-Bertani broth at 37°C and then diluted to 10^7^ CFU/mL in Luria-Bertani medium supplemented with AA at final a gradient of concentrations ranging from 50 μM and 100 μM. A separate Luria-Bertani culture without the AA was set as the control. Next, 200 μL of the aforementioned cultures with or without AA was transferred to each well of a 96-well, microtiter plates, which were then incubated at 37°C. Growth was again measured by taking OD readings at 600 nm at 24 h.

### Network pharmacology analysis

HERB (High-throughput Experiment-and Reference-guided database of traditional Chinese medicine), HIT (Herbal Ingredients’ Targets Platform), STITCH (Search Tool for Interactions of Chemicals, combined score ≥ 0.8), TargetNet (Probability ≥ 0.8), and NetInfer (Score ≥ 2) were applied to search or predict potential targets. Search Tool for the Retrieval of Interacting Genes/Proteins (STRING) platform was used to construct the target interaction network and to perform functional annotation analysis.

### Transcriptome sequencing

Total RNA was extracted from colon tissue using TRIzol Reagent according to the manufacturer’s instructions (Invitrogen, California, USA), and genomic DNA was removed using deoxyribonuclease I (TaKara, Tokyo, Japan). Then, RNA quality was determined by 2100 Bioanalyzer (Agilent, California, USA) and quantified using the ND-2000 (NanoDrop Technologies, Massachusetts, USA). Sequencing was performed by Tianjin Novogene Co., Ltd. (Tianjin, China). Briefly, 1 μg of total RNA was used for reverse transcription into cDNA. Then, A 200-300 bp cDNA target fragment was amplified for 15 RNA cycles, which was used to construct a cDNA library. After quantification by TBS380, the paired-end RNA-seq library was sequenced on the Illumina NovaSeq 6000 (2 × 150 bp read length).

### Transcriptional module repertoire analyses

RNA-seq data were initially filtered to obtain clean data. To identify differentially expressed genes (DEGs) between two different samples, the expression level of each transcript was calculated according to the fragments per kilobase of exon per million mapped reads method. Gene ontology (GO) and Kyoto encyclopedia of genes and genomes (KEGG) enrichment analysis were performed on the genes associated with the core targets using the Database for Annotation, Visualization and Integrated Discovery (DAVID) (https://david.ncifcrf.gov/). The species was set to “Mus musculus”, the p-value was set to “< 0.05”.

### Statistical analysis

Statistical analyses were conducted using GraphPad Prism software (version 6). Whenever applicable, the one-way analysis of variance (ANOVA) followed by LSD-t test for multiple comparisons was used. All values are presented as mean ± S.D. *p* < 0.05 is considered statistically significant.

## Results

### AA ameliorates colon damage in mice caused by the *S.T* infection

**Figure 1:**
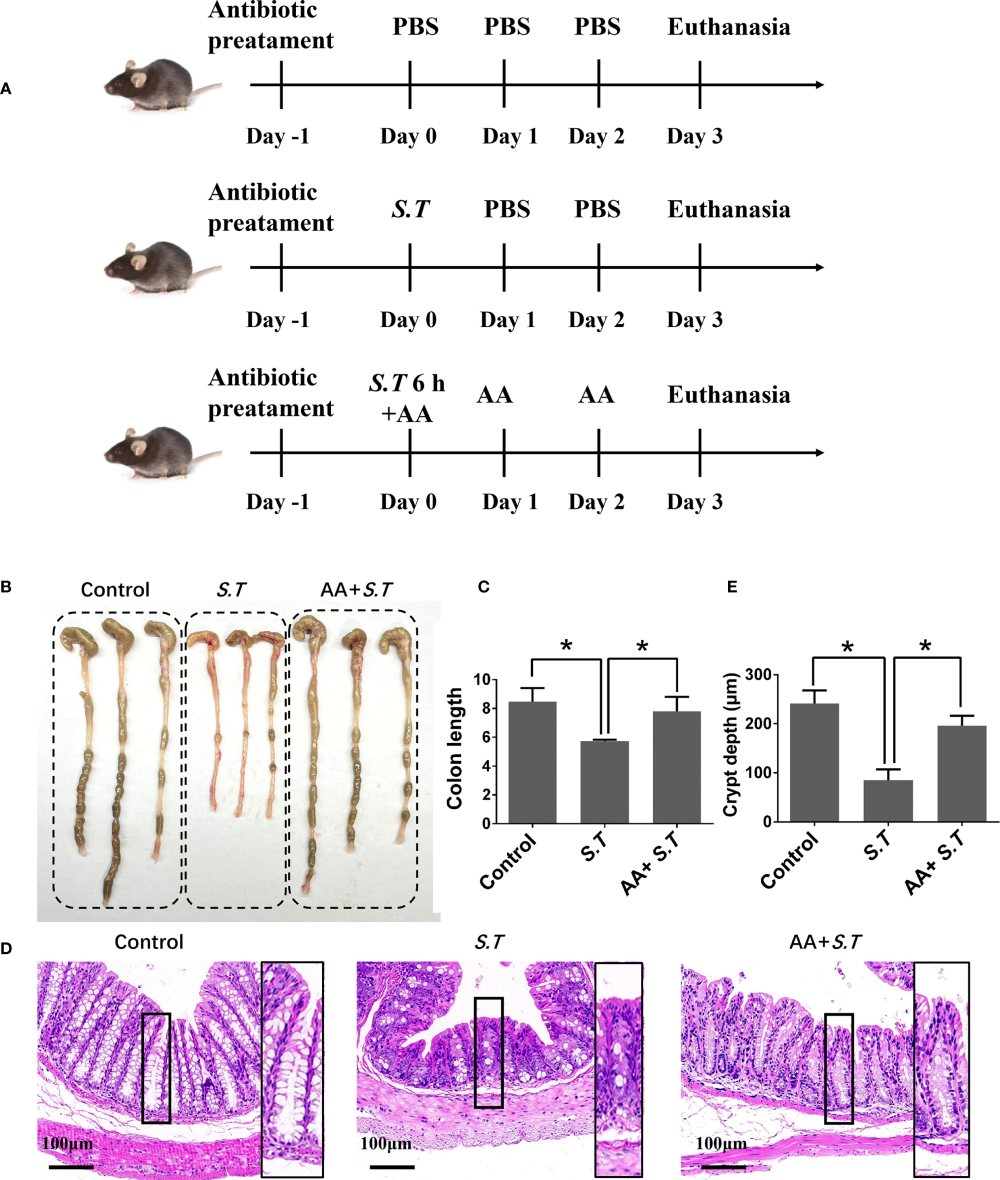
Effect of AA on *S.T*-induced colonic injury. SPF mice were administered *S.T* by oral gavage (1 × 10^9^ CFU/mouse) to model *S.T* infection. AA (10 mg/kg) was gavage administered to mice 6 h after the *S.T* infection. **(A)** Protocol for animal infection and drug delivery. **(B)** Colon macro morphology after the *S.T.* infection with or without AA treatment. **(C)** Measurement of colon length (n = 3). **(D)** Hematoxylin-eosin (H&E) stained colon sections after the *S.T.* infection with or without AA treatment. **(E)** Colon crypt depth (n = 10). Data are presented as mean ± standard deviation *, *p* < 0.05.

*S.T* infection can quickly trigger an inflammatory response that modifies the intestinal environment and disrupts the intestinal barrier (38). Mice were infected with wild-type *S.T* by daily oral administration of 1 × 10^9^ CFU for 3 days (**Figure 1A**). As expected, the length of the colon was reduced by *S.T* infection (**Figures 1B, C**). To observe the microstructure of the colon, H&E staining was performed, which showed that the crypt depth was significantly reduced in the *S.T*-infected mice (**Figures 1D, E**). These data confirm that *S.T* causes significant damage to the colon.

Dextran sulfate sodium-induced colon damage is alleviated by AA (33). Therefore, we hypothesized that a similar response to the *S.T*-induced colon injury may occur after AA administration. As predicted, AA significantly increased the length of the colon reduced by *S.T* infection (**Figures 1B, C**) and increased crypt depth (**Figures 1D, E**). Collectively, these results indicate that *S.T*-induced colon damage is alleviated by AA.

### AA restores the impaired colonic barrier in the *S.T*-infected mice

**Figure 2:**
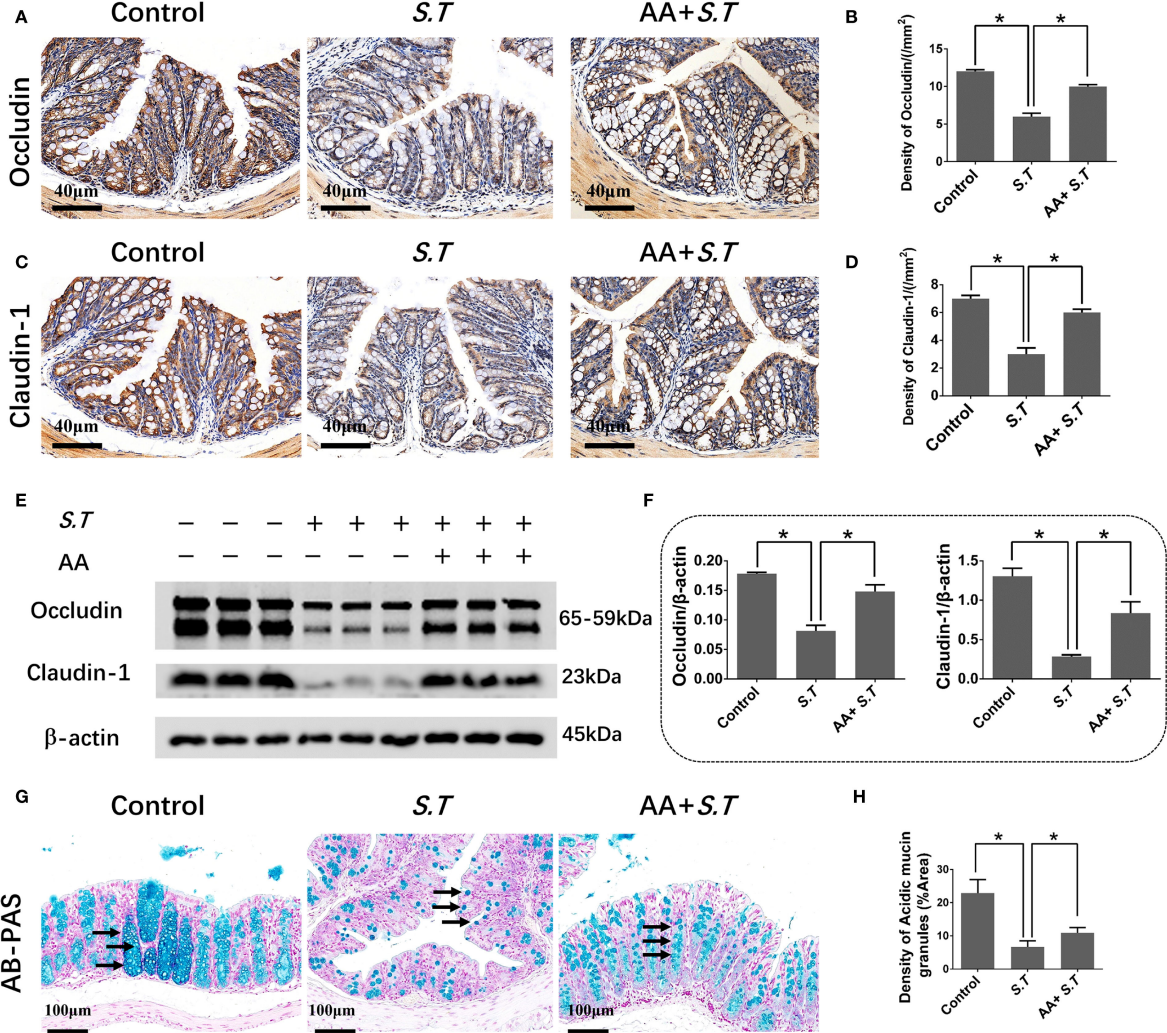
Effect of AA on *S.T*-induced barrier damage. **(A–D)** Immunohistochemistry (IHC) analysis of occludin and claudin 1 levels in colons of different treatment groups (n = 5). **(E, F)** Western blot analysis shows the levels of occludin and claudin 1 in the *S.T*-infected colon tissues with or without AA treatment (n = 3). **(G)** AB-PAS staining showing acidic mucin granule levels in the *S.T*-infected colons with or without AA treatment. Mucin is indicated by the black arrow (n = 5). **(G)** AB-PAS staining analysis shows the level of acidic mucin granules of colon infected by the *S.T* with or without AA treatment. Acidic mucin granules are indicated by the black arrow. **(H)** Measurement of acidic mucin granules levels (n = 5). Data are presented as mean ± standard. *, *p* < 0.05.

The intestinal mucosal barrier is a crucial line of defense against pathogens. The strength of this barrier is maintained by the integrity and tight connections of IECs. Therefore, we assessed the level of EPCAM, an IEC marker, in the colon of mice by immunohistochemistry. We observed that the level of EPCAM in the colon was not affected by *S.T* infection (**Supplementary Figure S1**). Next, IHC and western blotting was used to examine the levels of tight junction proteins. *S.T* infection significantly decreased the levels of occludin and claudin 1 compared with those in the control group (**Figures 2A–F**). Furthermore, AA significantly increased the levels of occludin and claudin 1 compared to those in the *S.T*-infected mice (**Figures 2A–F**). IECs are often covered with a layer of mucin secreted by goblet cells, which blocks the penetration of harmful substances and chemicals (39). We therefore performed AB-PAS staining to quantify the levels of acidic mucin granules. Not surprisingly, *S.T* infection significantly reduced the level of acidic mucin granules in the colon (**Figures 2G, H**). Conversely, AA significantly increased the level of acidic mucin granules (**Figures 2G, H**). These results indicate that AA alleviates *S.T-*induced damage to the colonic barrier.

### AA affects ISC activity by inhibiting the Notch2 pathway

**Figure 3:**
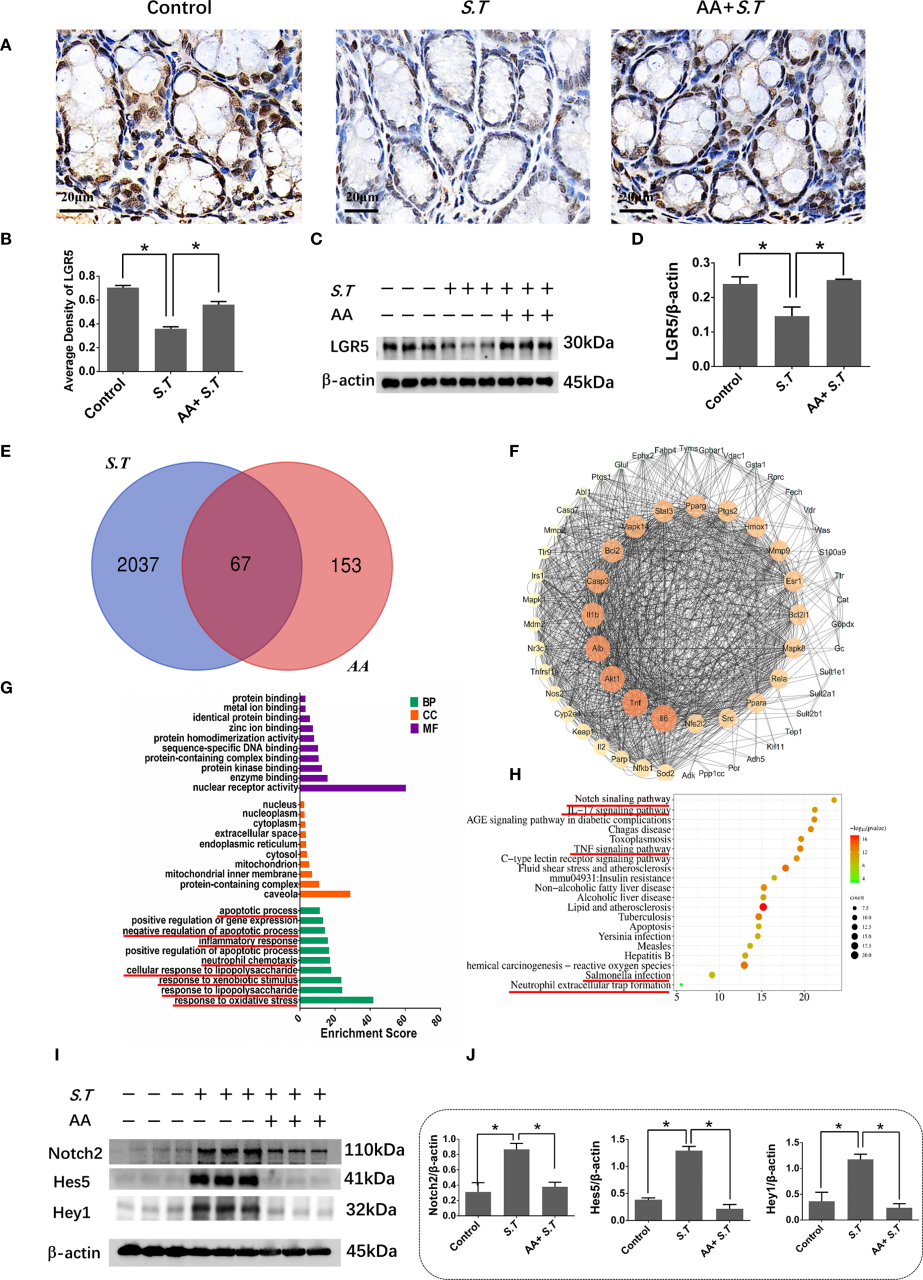
Effect of AA on *S.T*-induced activation of ISCs and the Notch2 pathway **(A, B)**. IHC analysis of LGR5 levels in colons of different treatment groups (n = 5). **(C, D)** Western blot analysis of LGR5 levels in the *S.T*-infected colon with or without AA treatment (n = 3). **(E)** Venn diagram shows the AA targets and the *S.T* infection-related genes. **(F)** Protein-protein interaction (PPI) network of 67 AA- and *S.T* infection-related targets. Node size is proportional to its degree. **(G)** The Gene Ontology (GO) database was used to classify the intersecting targets according to BP (Biological Process), CC (Cellular Component), and MF (Molecular Function). **(H)** KEGG Pathway enrichment of intersecting targets. **(I, J)** Western blot analysis of Nothc2, Hes5, and Hey1 levels in the *S.T*-infected colon with or without AA treatment (n = 3). Data are presented as mean ± standard. *, *p* < 0.05.

*S.T* infection inactivates ISCs (40); therefore, to observe ISC activation, we performed immunohistochemistry and western blotting for LGR5, a highly specific marker for ISCs located above crypts (41). The results showed significantly reduced levels of LGR5 in the *S.T*-infected mice (**Figures 3A–D**). AA significantly increased the levels of LGR5 compared with the levels in the *S.T*-infected mice (**Figures 3A–D**). These data support the reversal of *S.T.*-induced ISC inactivity by AA.

To explore the mechanism by which AA ameliorates *S.T* infection, network pharmacology analysis was performed. We obtained 2104 predicted disease targets and 220 drug targets from databases (**Figure 3E**). Among these, 67 AA- and *S.T* infection-related targets were considered hub targets (**Figure 3E**). A protein-protein interaction (PPI) network containing 20 nodes and 42 edges was constructed that included IL6, TNF, IL1B, IL2, PTGS1, and NFBκ1 (**Figure 3F**). GO (**Figure 3G**) and KEGG (**Figure 3H**) analyses were then performed using the DAVID. In biological processes (BP), hub targets were majorly enriched in response to xenobiotic stimulus, response to oxidative stress, apoptotic process, inflammatory response, cellular response to lipopolysaccharide, and response to lipopolysaccharide. Among 20 KEGG-enriched pathways were the Notch, IL17, and TNF signaling pathways, and *Salmonella* infection.

Overactivation of the Notch pathway inhibits the differentiation of ISCs (42, 43), and the Notch pathway was significantly enriched in the network pharmacology analysis (**Figure 3H**). To understand the role of the Notch2 pathway in *S.T*-infected mice, the levels of related proteins were determined using western blotting. The levels of Notch2, Hes5, and Hey1 were significantly increased in the *S.T* infected colon (**Figures 3I, J**). AA significantly decreased the levels of Notch2, Hes5, and Hey1 compared with those in the *S.T*-induced mice (**Figures 3I, J**). These results indicate that the *S.T*-activated Notch2 pathway is inhibited by AA.

### The transcriptome signature of *S.T*-infected colon samples is associated with neutrophils

**Figure 4:**
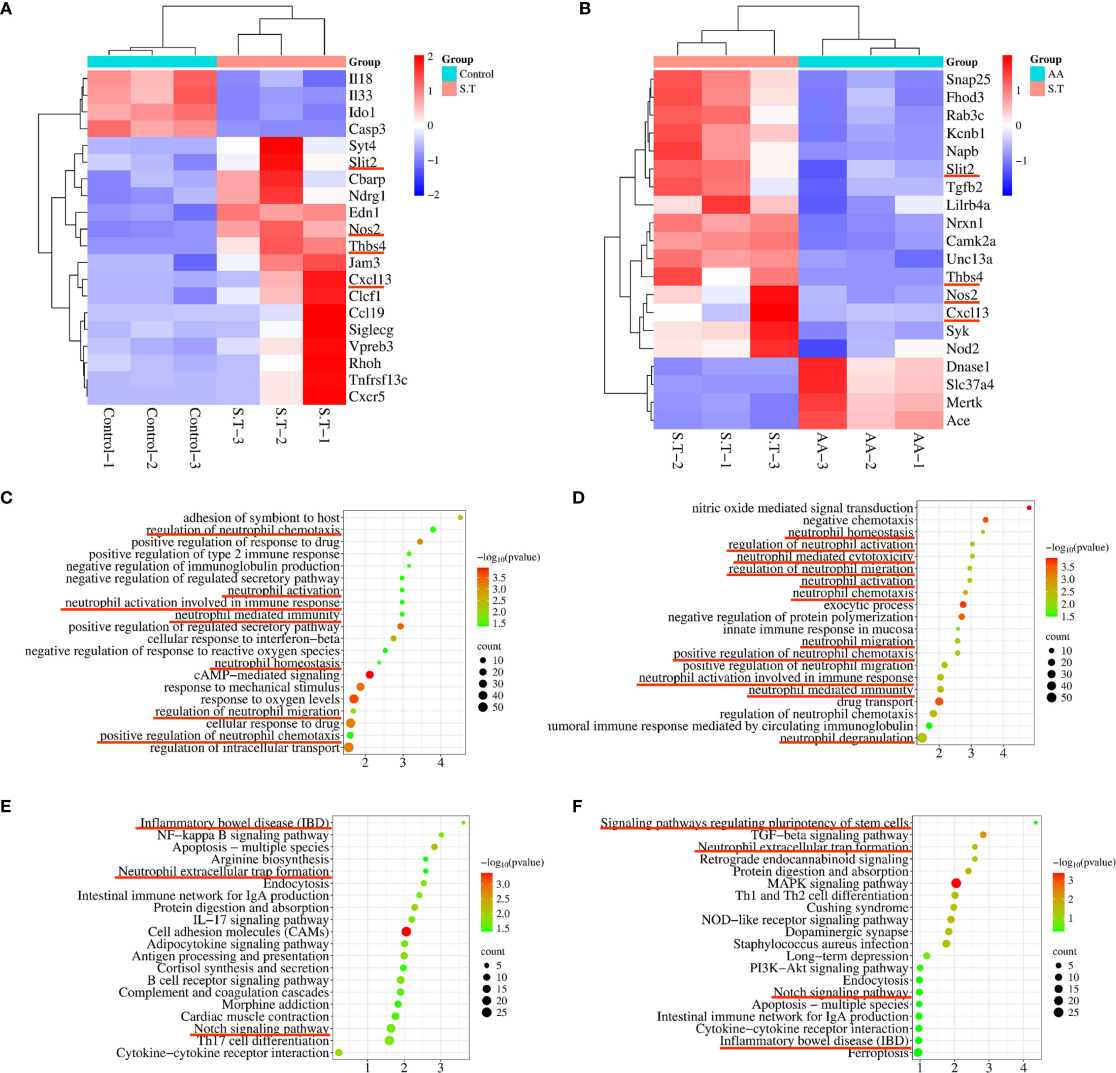
Transcriptome analysis to determine the critical role of neutrophils in the AA treatment of *S.T* colitis. Heatmap showing differentially expressed genes (DEGs) associated with neutrophils. **(A)** The control compared with the *S.T* infection. **(B)**The *S.T* infection compared with the AA treatment. Bubble map diagrams display the 20 most significant GO enrichment terms. **(C)** The control compared with the *S.T* infection. **(D)** The *S.T* infection compared with the AA treatment. Bubble map diagrams display the 20 most significant GO enrichment terms. **(E)** The control compared with the *S.T* infection. **(F)** The *S.T* infection compared with the AA treatment. *, *p* < 0.05.

To study the targets and signaling pathways involved in the AA-amelioration of *Salmonella* colitis, we performed transcriptome analysis. This analysis highlighted the involvement of neutrophils. DEGs involved in processes related to neutrophils including *Slit2*, *Thbs4*, *Nos2*, and *Cxcl13* were significantly upregulated after the *S.T* infection (**Figure 4A**). Conversely, these genes were significantly downregulated after the AA treatment (**Figure 4B**). Next, GO and KEGG enrichment analysis were then performed on the DEGs. The results show that the DEGs following the *S.T* infection and the AA intervention were enriched in multiple neutrophil-related pathways, including neutrophil homeostasis, neutrophil activation involved in immune response, neutrophil mediated immunity, neutrophil activation, regulation of neutrophil chemotaxis, regulation of neutrophil migration, regulation of neutrophil migration, neutrophil activation involved in immune response, neutrophil mediated immunity, and neutrophil degranulation (**Figures 4C–F**). Therefore, we speculate that neutrophils play an important role in AA-induced colonic damage caused by *S.T* infection.

### The protective effect of IECs induced by AA depends on neutrophils

**Figure 5:**
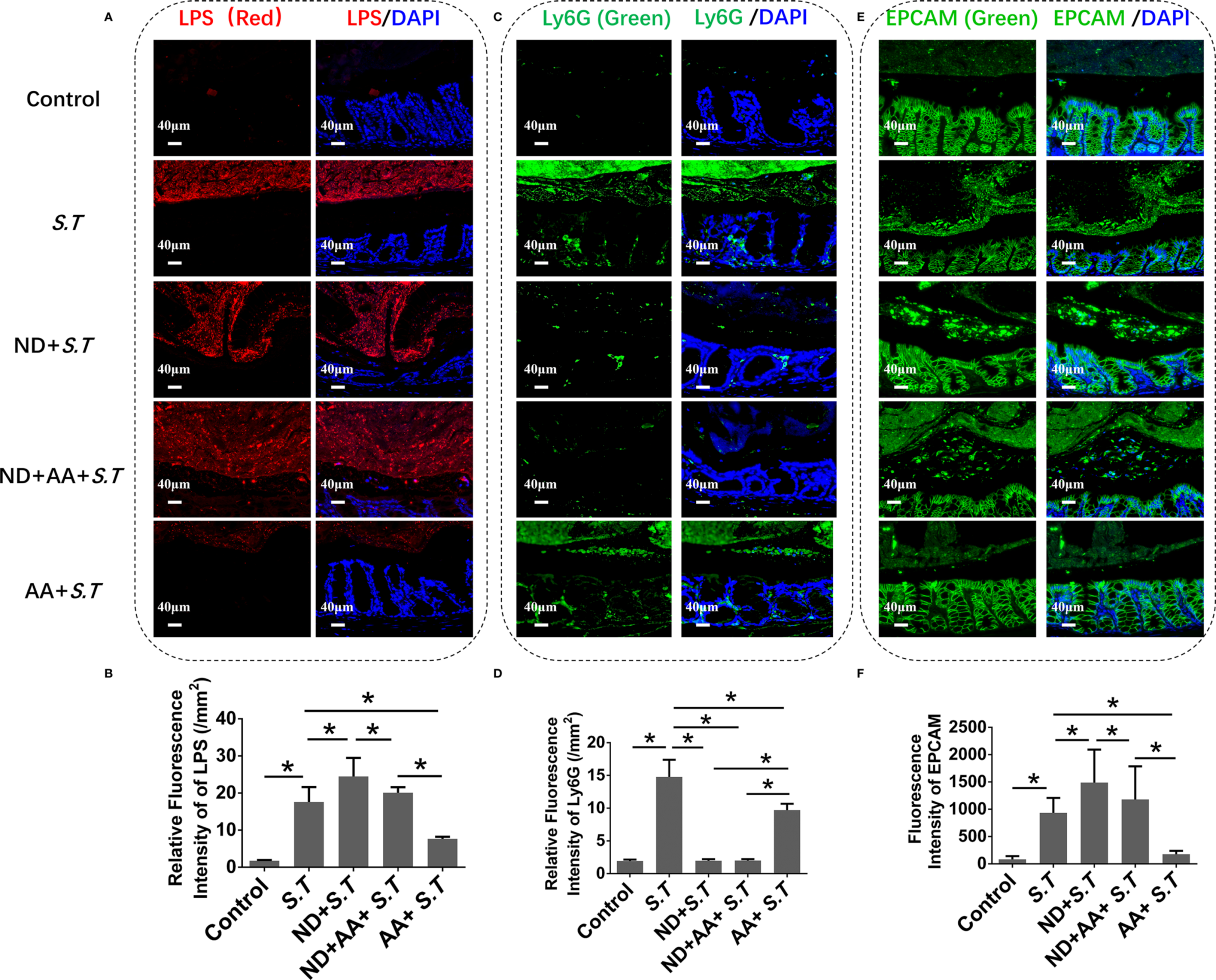
Effect of neutrophil depletion on the regulation of luminal *S.T* loads and IEC expulsion by AA. SPF mice were administered *S.T* by oral gavage (1 × 10^9^ CFU/mouse) to model *S.T* infection. AA (10 mg/kg) was gavage administered to mice 6 h after *S.T* infection. SPF mice were neutrophil depleted by daily intraperitoneal injection of anti-mouse Ly6G-Purified (250 μg/mouse) for 3 days. **(A)** IHC of LPS in colons of different treatment groups. **(B)** Fluorescence intensity of LPS signal in mouse colon (n = 5). **(C)** IHC of Ly6G in colons of different treatment groups. **(D)** Fluorescence intensity of Ly6G signal in mouse colon (n = 5). **(E)** IHC of EPCAM in colons of different treatment groups. **(F)** Fluorescence intensity of EPCAM signal in mouse colon (n = 5). Data are presented as mean ± standard. *, *p* < 0.05.

As an early line of defense against bacterial infections, neutrophils are recruited into the intestinal lumen during pathogen infection (32–34). Immunohistochemistry showed a significantly higher level of *S.T* around intestinal tissue in the *S.T*-infected mice than in the control mice (**Figures 5A, B**), which was colocalized with neutrophils (**Supplementary Figure S2**). Neutrophils were recruited into the intestinal lumen after the *S.T* infection, which forms a barrier preventing *S.T* invasion (**Figures 5C, D**). AA significantly reduced the levels of *S.T* and neutrophils around the colon tissue (**Figures 5A, B**), which may result from the phagocytosis of *S.T* by neutrophils. We then stained colon tissue for the IEC marker, EPCAM, which showed that the level of IEC efflux was significantly increased in the *S.T*-infected mice (**Figures 5E, F**). This was significantly attenuated by the AA intervention (**Figures 5E, F**), which may be related to the reduction in *S.T* counts. These results indicate that IEC efflux is inhibited by AA, which may be related to neutrophils.

To investigate this possibility further, mice were injected intraperitoneally with Ly6G, which significantly reduced the neutrophil numbers (**Supplementary Figure S3**; **Figures 5C, D**). Surprisingly, the large numbers of *S.T* were found around the colon tissue of the neutrophil-deficient mice (**Figures 5A, B**). AA did not reduce the level of *S.T* around the colon in the mice with neutrophil depletion (**Figures 5A, B**). After neutrophil depletion, the number of shed IECs increased (**Figures 5E, F**) compared with that in the *S.T*-infected group. Moreover, the inhibitory effect of AA on IEC shedding was attenuated after neutrophil depletion (**Figures 5E, F**). These results indicate that AA regulation of IEC shedding is associated with neutrophils.

Upon infection, neutrophils rapidly migrate to the intestinal tract and phagocytose pathogens. To demonstrate the relationship between AA and neutrophil phagocytosis of bacteria, HL-60 cells were cultured *in vitro* and stimulated with all-trans-retinoic acid to induce differentiation into neutrophils (44). The number of *S.T* outside the neutrophils was significantly reduced after the AA intervention by calculating the phagocytosis% *in vitro*. (**Supplementary Figure S4A**). Next, we found that AA significantly inhibited the growth of *S.T*. (**Supplementary Figure S4B**). These results demonstrate that AA enhances the phagocytic function of neutrophils. In summary, neutrophils play an important role in AA-mediated colonic damage caused by *S.T* stimulation.

### The effect of AA on ISC activity is dependent on neutrophils

**Figure 6:**
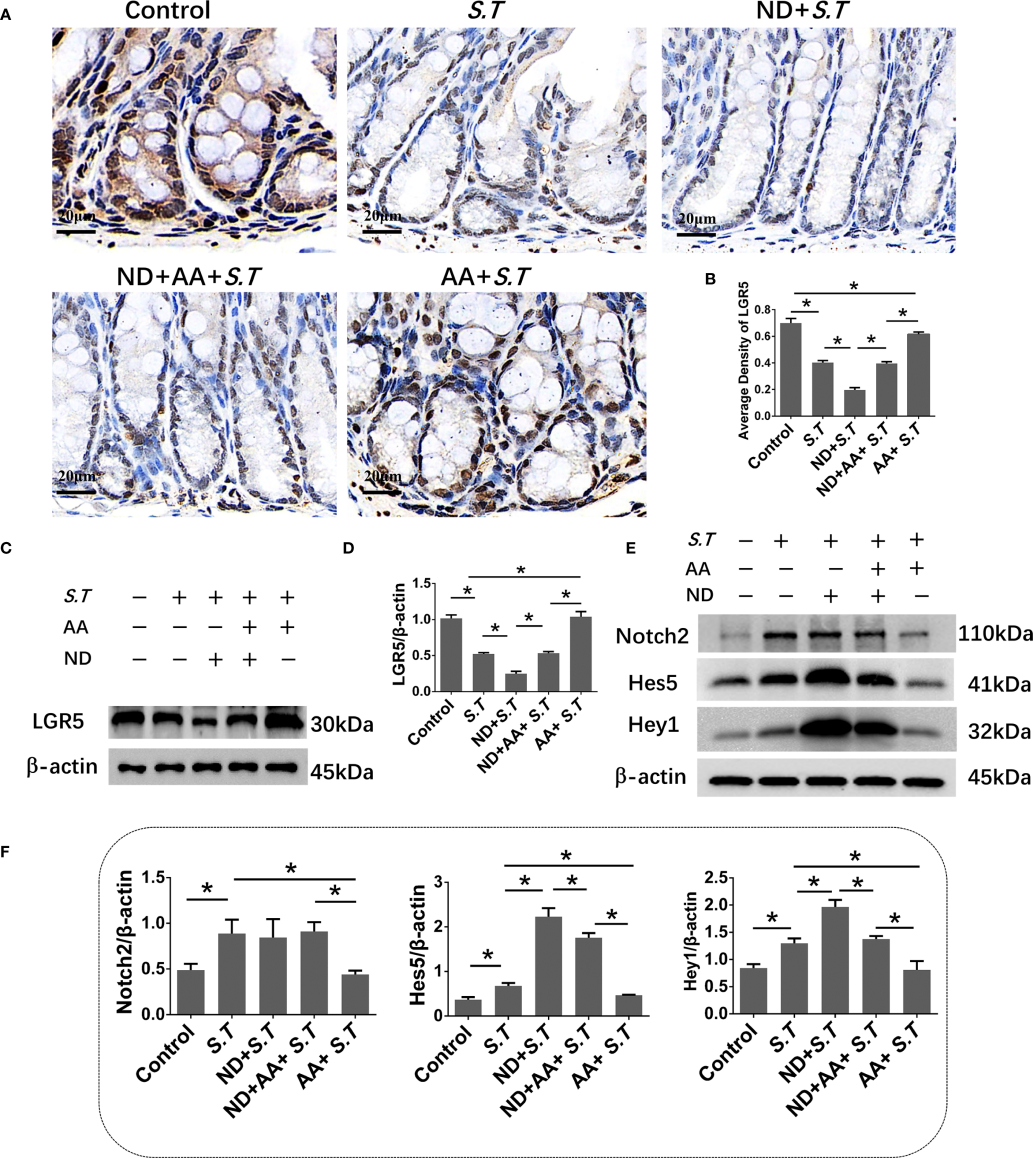
Effect of neutrophil depletion on the AA modulation of ISCs and the Notch2 pathway. **(A, B)** IHC analysis of LGR5 levels in colons of different treatment groups (n = 5). **(C, D)** Western blot analysis of LGR5 levels in colons of different treatment groups (n = 3). **(E, F)** Western blot analysis of the levels of Notch2, Hes5, and Hey1 in colons of different treatment groups (n = 3). Data are presented as mean ± standard. *, *p* < 0.05.

To further observe ISC activity in neutrophil-deficient mice, we examined levels of LGR5 by immunohistochemistry and western blotting. LGR5 levels were reduced in the neutrophil-deficient mice compared with those in the *S.T*-infected mice (**Figures 6A–D**). The effect of AA on ISC activity was significantly reduced after the neutrophil depletion (**Figures 6A–D**). These results demonstrated that the effect of AA on ISCs is dependent on neutrophils during the *S.T* infection. Next, western blotting was used to detect the levels of key proteins in the Notch2 pathway. As predicted, the levels of Hes5 and Hey1 were significantly increased in the neutrophil-deficient mice than in the *S.T*-infected mice (**Figures 6E, F**). The inhibitory effect of AA on the Notch2, Hes5 and Hey1 were reduced in the neutrophil-deficient mice (**Figures 6E, F**). These results demonstrate that AA influences the activity of ISCs by neutrophils via Notch2/Hes5/Hey1. In summary, the effect of AA on ISCs is dependent on neutrophils during the *S.T* infection.

### The protective effect of the intestinal mucosal barrier induced by AA is dependent on neutrophils

**Figure 7:**
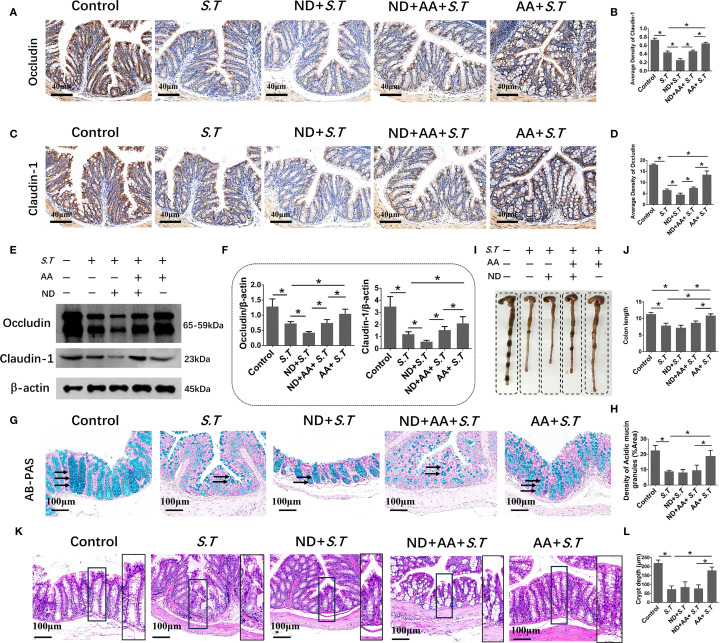
Effect of neutrophils in the alleviation of colonic injury by AA. **(A–D)** IHC of occludin and claudin 1 in colons of different treatment groups (n = 5). **(E, F)** Western blot analysis of occludin and claudin 1 levels in colons of different treatment groups (n = 3). **(G)** AB-PAS staining shows the levels of acidic mucin granules in colons of different treatment groups. Acidic mucin granules are indicated by the black arrow. **(H)** Measurement of acidic mucin granules levels (n = 5). **(I)** Colon macro morphology of different treatment groups. **(J)** Measurement of colon length (n = 3). **(K)** H&E staining of colon sections from different treatment groups. **(L)** Colon crypt depth (n = 10). Data are presented as mean ± standard. *, *p* < 0.05.

To better define the contribution of neutrophils to the maintenance of the intestinal mucosal barrier in the *S.T*-infected mice, IHC and western blotting were used to examine tight junction proteins. The levels of occludin and claudin 1 were significantly reduced in the colon tissues of neutrophil-deficient mice compared with those in the *S.T* group (**Figures 7A–F**). Meanwhile, neutrophil depletion significantly reduced the ability of AA to affect the low levels of claudin 1 and occludin caused by the *S.T* infection (**Figures 7A–F**). Similarly, the restorative effect of AA on acidic mucin granule levels (**Figures 7G, H**), colon length (**Figures 7I, J**) and crypt depth (**Figures 7K, L**) was reduced in the neutrophil-depleted mice. Moreover, the levels of occludin and claudin 1, acidic mucin granules and crypt length were slightly decreased in mice with isolated neutrophil depletion; however, there was no significant difference compared with the control group (**Supplementary Figure S5**). These results demonstrate that the effect of AA on *S.T* colitis is dependent on neutrophils.

## Discussion

In this study, we elucidated the protective effect of AA on *S.T*-induced colon injury in mice. *S.T* infection damages the intestinal mucosal barrier, which is associated with the expulsion of IECs. IEC efflux was significantly changed after AA intervention. Neutrophil depletion exacerbated *S.T* infection by reducing the activation of ISCs through the suppression of Notch2 signaling. We also demonstrated that the reparative effect of AA on *S.T*-induced intestinal mucosal barrier damage decreased after neutrophil depletion.

Accumulating evidence indicates that *S.T* actively invades IECs and survives in *Salmonella* cysts and the cell cytosol (10, 45). In the cytoplasm, *S.T* replicates rapidly, expresses invasion factors, and triggers the extrusion of infected IECs into the intestinal lumen (10). Our data showed that a large number of IECs were expelled into the intestinal lumen after the *S.T* infection. After AA intervention, fewer IECs were expelled into the intestinal lumen and the damage to colon tissue was reduced, showing that AA inhibited *S.T*-induced colon damage.

Colon injury is usually accompanied by intestinal mucosal barrier dysfunction, with mucin and tight junctions being important indicators of intestinal mucosal barrier integrity (46, 47). Evidence from clinical and animal studies indicates that several intestinal inflammatory conditions, including inflammatory bowel disease, colon cancer, and numerous intestinal infections, are associated with significant changes in the levels of mucins and tight junction proteins (48). In the present study, the protective effect of AA on *S.T*-induced damage to the colonic mucosal barrier was confirmed.

Neutrophils are the most common immune cell type in the circulatory system and are an important line of defense against microbial infections (22, 49). They provide potent defense against intestinal infections using a variety of effector mechanisms, such as nitrate species, antimicrobial peptides, and neutrophil extracellular traps (50–52).

Neutrophils are recruited into the intestinal lumen during intestinal microbial invasion and form a physical defense that prevents direct contact between microbes and intestinal tissue (53). Molloy et al. found that neutrophils can form intraluminal casts to prevent the spread of pathobionts to systemic organs in *Toxoplasma gondii*-infected mice (54). Our data showed that neutrophils were recruited to the intestinal lumen after the *S.T* infection and were in close contact with secreted *S.T* in the intestinal lumen, resembling the behavior of neutrophils that form a physical barrier upon infection (54).

Upon neutrophil depletion, colon tissue was attacked by *S.T*, and more IECs were expelled into the intestinal lumen, demonstrating the crucial role of neutrophils in combating *S.T* infection. AA inhibited the *S.T*-induced recruitment of neutrophils and the expulsion of IECs. However, the protective effect of AA was significantly reduced after neutrophil depletion, confirming that AA inhibits *S.T* infection by affecting neutrophils.

Immune cells are involved in intestinal regeneration (21, 55). Inflammatory cytokines, such as IL-10, IL-22, and IL-13, are secreted by various immune cells and act on ISCs to promote IEC regeneration (21, 25, 55). Zhang et al. found that the immune response of neutrophils and the differentiation of ISCs were enhanced by chronic psychological stress (56), but there is no direct evidence of the relationship between neutrophils and ISCs. In this study, we found that the levels of LGR5-positive ISCs were significantly reduced after the *S.T* stimulation. Interestingly, neutrophil depletion suppressed ISC activity, indicating that neutrophils inhibit *S.T* infection by regulating ISCs. The Notch pathway is essential for the development, differentiation, proliferation, and apoptosis of ISCs (17, 18, 57, 58). Notch pathway activation inhibits the differentiation of ISCs into goblet cells and compromises the integrity of the intestinal mucosal barrier (59). Our results confirmed the effect of *S.T* infection on Notch2 pathway activation. AA inhibited Notch2 pathway activation and restored the activity of LGR5-positive ISCs, which was associated with neutrophils.

Neutrophils are mainly distributed in the circulatory system and are recruited in large numbers to colon tissue during *S.T* invasion, where they protect the colon by phagocytosing the bacteria. After neutrophil depletion, large numbers of *S.T* bacteria invade the colon, further damaging the intestinal barrier and reducing the levels of tight junction proteins, thereby increasing the risk of mortality (data not shown). In this study, AA effectively alleviated *S.T*-induced colitis through two mechanisms. On the one hand, AA intervention reduced the *S.T* counts, thereby blocking bacterial invasion of the colon. On the other hand, AA promoted the phagocytosis of *S.T* by neutrophils. When neutrophils were depleted, the protective effect of AA on the intestinal barrier significantly decreased, indicating that neutrophils are the key cells in the inhibition of *S.T*-induced colitis by AA. The roles of immune cells other than neutrophils may also be important in AA resistance to *S.T* infection and warrant investigation.

## Conclusion

This study reveals a previously unrecognized mechanism by which AA mitigates *S.T*-induced colitis through coordinated regulation of neutrophil dynamics and Notch2 signaling. We demonstrate that AA restores intestinal barrier integrity by suppressing Nothc2/Hes5/Hey1 activation, thereby rescuing LGR5^+^ ISC proliferation, while simultaneously modulating neutrophil recruitment to limit pathogen invasion and epithelial shedding. Crucially, neutrophil depletion decreased the therapeutic efficacy of AA, establishing its dependence on neutrophil-mediated immune regulation. These findings resolve an aspect of *S.T* pathogenesis by defining a “Neutrophil-Notch2-ISC” axis as an important means of mucosal repair. Therapeutically, this work positions AA as a dual-action agent capable of targeting both inflammatory and regenerative pathways, a paradigm shift from conventional anti-inflammatory strategies. Methodologically, the integration of a neutrophil-specific depletion model with a multi-omics approach provides a new framework for screening interventional *S.T-*induced colitis drugs. Our results underscore the potential of natural compounds to fine-tune host-pathogen interactions without compromising endogenous repair mechanisms. From a translational perspective, these insights advocate for therapies that synergistically enhance barrier regeneration and immune containment, offering a blueprint for managing antibiotic-resistant enteric infections. Future studies exploring the efficacy of AA in humanized models or combinatorial regimens with existing antimicrobials may accelerate its clinical translation.

## Data Availability

All relevant data is contained within the article: The original contributions presented in the study are included in the article/**Supplementary Material**, further inquiries can be directed to the corresponding author/s.
