# Supplementary material for: Unveiling the neutrophil-Notch2-ISC axis: asiatic acid’s therapeutic strategy in infectious colitis
Source: Front Immunol. 2025 Sep 22;16:1634063. doi: 10.3389/fimmu.2025.1634063 (PMC12497614; doi:10.3389/fimmu.2025.1634063)
Supplement: Supplementary file 1 [file DataSheet1.docx]

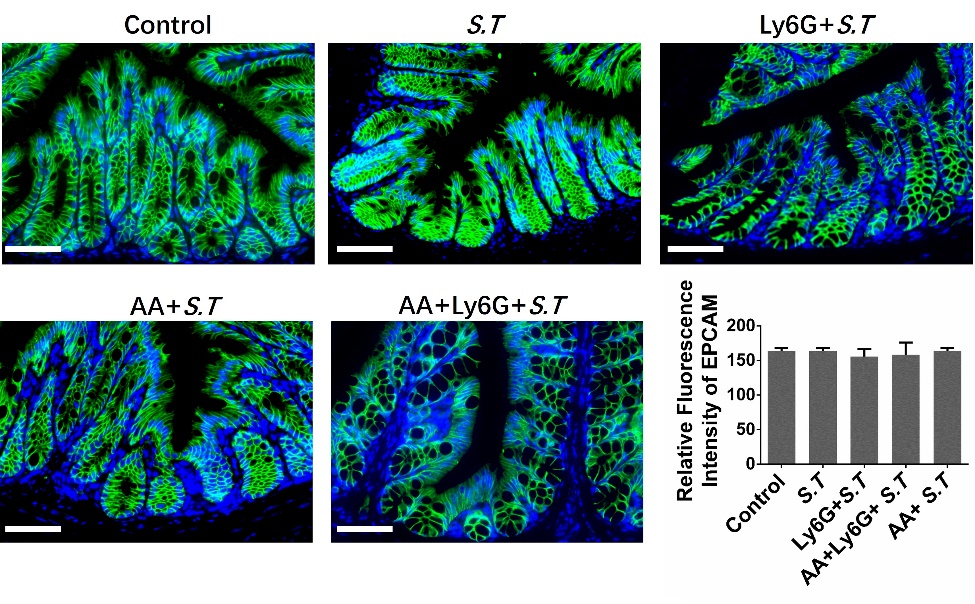


**Fig S1** Effect of neutrophil depletion on ECAPM protein expression in mice colon tissue. Mice were administered *S.T* by oral gavage (1 × 10^9^ CFU/mouse) to model *S.T* infection. AA (10 mg/kg) was gavage administered to mice 6 h after *S.T* infection. Mice were neutrophil depleted by daily intraperitoneal injection of anti-mouse Ly6G-Purified (250 μg/mouse) for 3 days. The expression of EPCAM protein levels were detected by IHC in different treatment groups of mice. Fluorescence intensity of EPCAM expression in mice colon (n = 5; **p*<0.05). Scale bars, 40 μm. Data are presented as mean ± standard deviation.


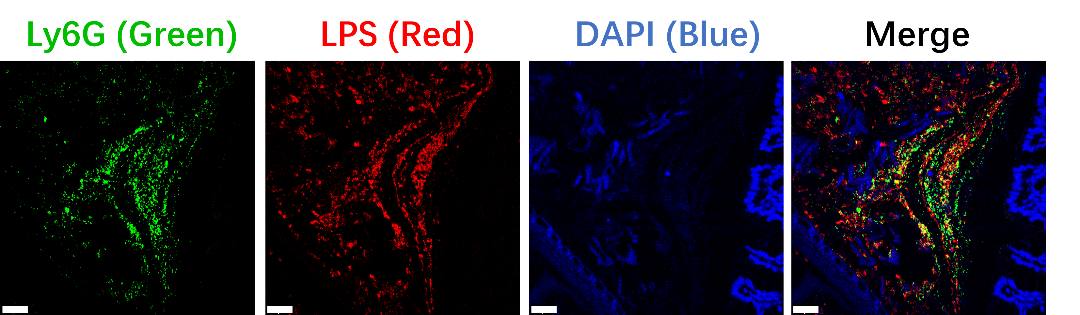


**Fig S2** Interactions between neutrophils and pathogens in the colon of infected mice. The expression of LPS (red) and Ly6G (green) protein levels were detected by IHC in different treatment groups of mice. Scale bars, 25 μm.


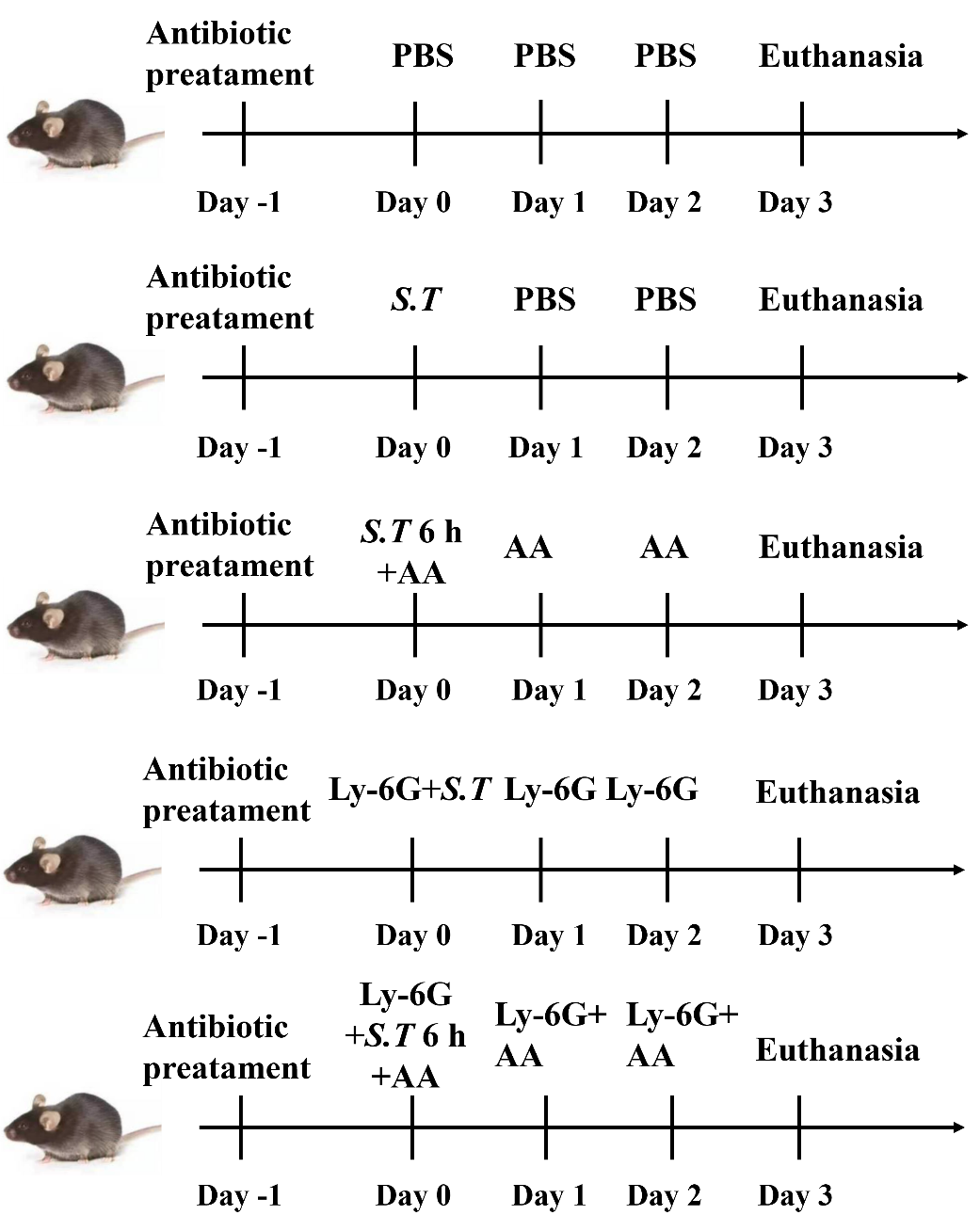


**Fig S3** Protocol for neutrophil-depleted mice.

**
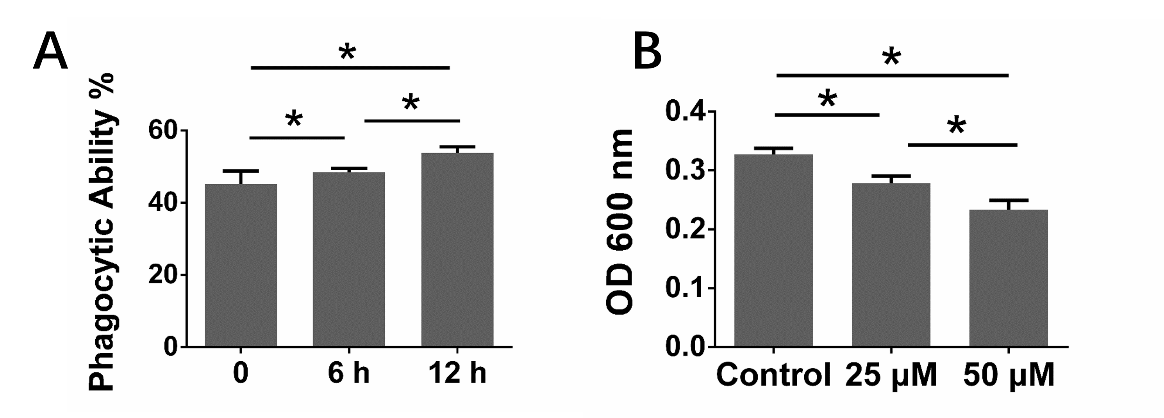
**

**S4** (A) The effect of AA on neutrophil phagocytosis of bacteria (n = 3). (B) The effect of AA on bacterial growth (n = 3). Data are presented as mean ± standard. *, *p* < 0.05.

**
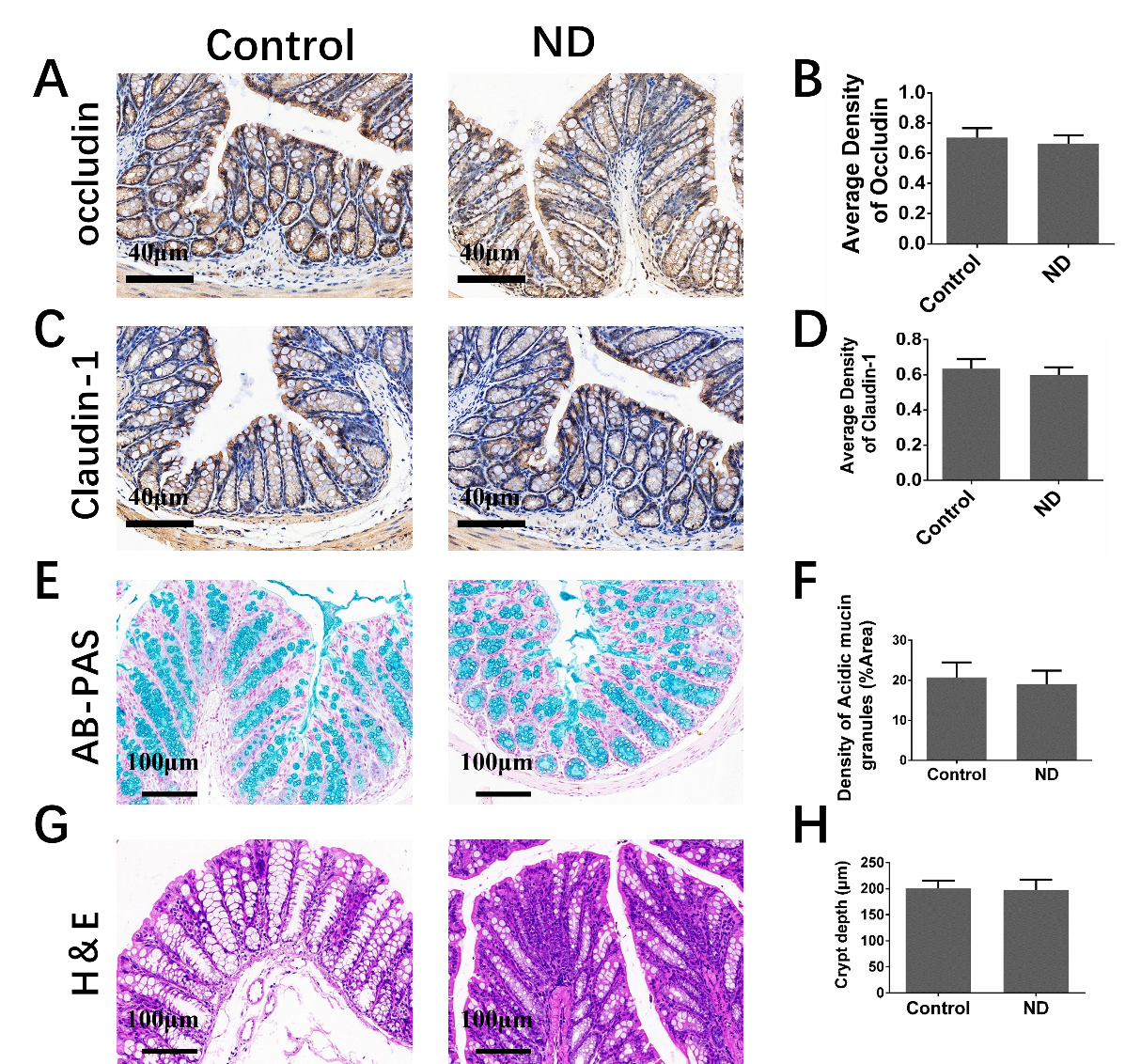
**

**S5 (**A–D**)** IHC of occludin and claudin 1 in colons of different treatment groups (n = 5). (E) AB-PAS staining shows the levels of acidic mucin granules in colons of different treatment groups. Acidic mucin granules are indicated by the black arrow. (F) Measurement of acidic mucin granules levels (n = 5). (G) H&E staining of colon sections from different treatment groups. (H) Colon crypt depth (n = 10). Data are presented as mean ± standard. *, *p* < 0.05.
